# A potential risk factor associated with acute tumor lysis syndrome in dogs with multicentric lymphoma receiving chemotherapy

**DOI:** 10.1093/jvimsj/aalaf088

**Published:** 2026-02-02

**Authors:** Hiroki Yamazaki, Konami Nagai, Yusuke Wada, Shunsuke Noguchi, Shushi Yamamoto, Toshikazu Sakai, Shidow Torisu

**Affiliations:** Companion Animal Internal Medicine, Department of Companion Animal Clinical Sciences, School of Veterinary Medicine, Rakuno Gakuen University, Ebetsu, Hokkaido, 069-8501, Japan; The Veterinary Medical Center, Graduate School of Life and Environmental Sciences, Osaka Metropolitan University, 1-58 Rinku-Oraikita, Izumisano 598-8531, Japan; Companion Animal Internal Medicine, Department of Companion Animal Clinical Sciences, School of Veterinary Medicine, Rakuno Gakuen University, Ebetsu, Hokkaido, 069-8501, Japan; Department of Oncology, Osaka Hospital, Japan Animal Referral Medical Center, 3-14-7 Senbanishi, Minoo-shi, Osaka 562-0036, Japan; The Veterinary Medical Center, Graduate School of Life and Environmental Sciences, Osaka Metropolitan University, 1-58 Rinku-Oraikita, Izumisano 598-8531, Japan; Department of Oncology, Osaka Hospital, Japan Animal Referral Medical Center, 3-14-7 Senbanishi, Minoo-shi, Osaka 562-0036, Japan; Companion Animal Veterinary Surgery, Department of Companion Animal Clinical Sciences, School of Veterinary Medicine, Rakuno Gakuen University, 582-1 Bunkyodai-Midorimachi, Ebetsu, Hokkaido 069-8501, Japan; Companion Animal Veterinary Surgery, Department of Companion Animal Clinical Sciences, School of Veterinary Medicine, Rakuno Gakuen University, 582-1 Bunkyodai-Midorimachi, Ebetsu, Hokkaido 069-8501, Japan; Companion Animal Veterinary Surgery, Department of Companion Animal Clinical Sciences, School of Veterinary Medicine, Rakuno Gakuen University, 582-1 Bunkyodai-Midorimachi, Ebetsu, Hokkaido 069-8501, Japan

**Keywords:** L-asparaginase, chronic kidney disease, metabolic acidosis, weight loss

## Abstract

**Background:**

Acute tumor lysis syndrome (ATLS) is caused by the rapid breakdown of tumor cells, leading to electrolyte imbalances and renal dysfunction. The risk of ATLS is particularly high in lymphoma, and therefore it is crucial to consider this risk when initiating chemotherapy. However, risk factors associated with ATLS in dogs remain largely unexplored.

**Hypothesis/Objectives:**

Identify potential risk factors for ATLS in dogs with multicentric lymphoma.

**Animals:**

A retrospective analysis was conducted on 24 dogs diagnosed with B-cell high-grade multicentric lymphoma that received chemotherapy.

**Methods:**

Blood samples were collected before treatment and on days 3, 5, and 8 after treatment. Serum concentrations of uric acid, potassium, phosphorus, calcium, and creatinine were measured based on the Cairo-Bishop Criteria to classify cases as laboratory tumor lysis syndrome (LTLS) or clinical tumor lysis syndrome (CTLS). Clinical variables, including signalment, clinical signs, comorbidities, stage, chemotherapy agents, hematologic and biochemical findings, chemotherapy response, and clinical outcomes were compared between two groups: normal and LTLS/CTLS.

**Results:**

Of 24 dogs, LTLS occurred in 5 (20.8%), whereas CTLS occurred in 2 (8.3%). The development of LTLS/CTLS was strongly associated with the initial administration of L-asparaginase, presence of chronic kidney disease (CKD), weight loss, and metabolic acidosis. However, no significant differences were observed between the normal and LTLS/CTLS groups regarding signalment, clinical signs, stage, CBC abnormality, and clinical outcomes.

**Conclusions and clinical importance:**

Evaluating the four risk factors at the initiation of chemotherapy may help establish personalized prevention strategies for ATLS in dogs with multicentric lymphoma.

## Introduction

Acute tumor lysis syndrome (ATLS) is a critical oncologic emergency that results from the rapid lysis of malignant cells after chemotherapy or radiation therapy. The destruction of malignant cells leads to the release of intracellular contents, including phosphorus, potassium, and purines, into the systemic circulation. This sudden release exceeds the excretory capacity of the kidneys, resulting in severe metabolic and electrolyte disturbances.^1^ Lymphoma cells have higher demands for nucleic acids and adenosine triphosphate (ATP), resulting in a higher phosphorus content compared with normal lymphocytes.^2^ Consequently, the rapid breakdown of these tumor cells results in marked hyperphosphatemia and subsequent hypocalcemia because of calcium phosphate precipitation within renal tubules, potentially culminating in oliguric or anuric acute renal failure.^3,4^ Clinical manifestations of hypocalcemia may include tetany, cardiac arrhythmias, syncope, or seizures.^3,4^ Furthermore, release of intracellular potassium during tumor cell lysis may provoke hyperkalemia, presenting as lethargy, weakness, bradycardia, syncope, or life-threatening cardiac arrhythmias.^3,4^ Therefore, it is crucial to consider this risk when initiating chemotherapy. However, the timing of ATLS onset is difficult to predict, and risk factors associated with ATLS in dogs remain largely unexplored.

We retrospectively investigated potential risk factors for ATLS in dogs with high-grade multicentric lymphoma treated with chemotherapy. Given that ATLS is more likely to occur shortly after the initial induction of chemotherapy,^1,5^ blood samples were collected and analyzed within one week of the initial induction. We hypothesized that several risk factors would be associated with ATLS in dogs with lymphoma.

## Materials and methods

### Medical record review and patient selection

We employed a retrospective design. Medical records from the secondary veterinary medical facilities at Osaka Metropolitan University were reviewed for dogs with B-cell high-grade lymphoma that was histopathologically or cytologically diagnosed and treated between 2017 and 2022. Clinical data collected included history, signalment, clinical signs at presentation, stage, clinicopathologic findings, diagnostic imaging, chemotherapy protocols, chemotherapy response, and clinical outcome. Permission to use clinical data was obtained by informed consent from the owners at the time of first medical examination. The clinical information also included records of regular follow-up communications with owners and referring veterinarians. The inclusion criteria for our study were: a confirmed diagnosis of multicentric lymphoma by cytology or histopathology or both; receipt of first-line chemotherapy for lymphoma; B-cell immunophenotype confirmed by either PCR for antigen receptor gene rearrangements or immunohistochemistry; treatment-naïve status (no prior chemotherapy, corticosteroids or other anticancer interventions); and, absence of concurrent neoplastic disease. Exclusion criteria were: any anticancer treatment before diagnosis, non-multicentric lymphoma subtypes, and failure to undergo follow-up laboratory testing at the defined post-treatment time points. All dogs underwent venous blood gas (VBG) analysis, and blood biochemical analysis, including complete blood count (CBC), and metabolic, renal and hepatic function testing at their first medical examination. The VBG analysis evaluated pH, PCO_2_, bicarbonate (HCO_3_), lactate (Lac), and base excess (BE) based on previously reported reference values for dogs.^6^ All dogs underwent hepatic and splenic ultrasonography, and fine-needle aspiration of target lesions was performed where indicated. Dogs with multicentric lymphoma were assigned a clinical stage (I–V) and substage (a/b) according to the World Health Organization (WHO) staging system.

### Chemotherapy protocol and response

All dogs with lymphoma were treated using multi-drug protocols based on L-asparaginase combined with cyclophosphamide, doxorubicin, vincristine sulfate, and prednisolone (L-CHOP) or cyclophosphamide, doxorubicin, vincristine sulfate, and prednisolone (CHOP), followed by rescue treatment protocols such as nimustine hydrochloride or lomustine (CCNU) with or without prednisone. All dogs were assessed for tumor response by ultrasonography or radiography or both every 2 weeks during chemotherapy in accordance with the response evaluation criteria in solid tumors (RECIST: v1.0) criteria.^7^ All dogs were assessed for early adverse events within the first 8 days after chemotherapy initiation and graded according to the Veterinary Cooperative Oncology Group–Common Terminology Criteria for Adverse Events criteria.^8^ Response was classified as a complete response (CR) when the disappearance of all measurable disease was documented, partial response (PR) when a reduction of at least 30% in the mean sum of the longest diameter of all target lesions was observed, progressive disease (PD) when a 20% increase in the mean sum of the longest diameter was detected, and stable disease (SD) when there was neither sufficient decrease to qualify for PR nor sufficient increase to qualify for PD. The overall response rate (ORR) was calculated as the percentage of dogs that achieved the best overall response of CR or PR. When the dogs exhibited chemotherapy failure, they received palliative or end-of-life care. Clinical outcomes documented in the medical records were used to determine progression-free survival (PFS) and overall survival (OS). The PFS was defined as the period from the first day of chemotherapy until the date of confirmed tumor progression, and OS was defined as the period from the first day of chemotherapy until the endpoint, which recorded all deaths.

### Diagnosis of tumor lysis syndrome

Serum biochemical tests were performed using cryopreserved serum collected from all dogs at their first medical examination before chemotherapy and on days 3, 5, and 8 after the initial induction of L-CHOP or CHOP. Because most TLS events have been reported to occur within 12–72 hours after treatment,^9^ with a peak at 48–72 hours, we standardized the initial evaluation on day 3. Preanalytically labile analytes were evaluated within 1 hour after sample collection in accordance with guidance from the American Society for Veterinary Clinical Pathology. Residual serum was stored at −80°C following a unified standard operating procedure. Deferred assays were limited to analytes with proven stability at −80°C for up to 12 months. Serum concentrations of uric acid (UA), potassium (K), phosphorus (P), calcium (Ca), and creatinine (Cre) were measured using a FUJI DRI-CHEM IMMUNO AU10V analyzer (Fujifilm Medical, Tokyo, Japan) to classify the cases as laboratory tumor lysis syndrome (LTLS) or clinical tumor lysis syndrome (CTLS) according to the Cairo–Bishop criteria for TLS, using relative changes of ≥25% (Table 1).^3^ The dogs then were divided into two groups: normal and LTLS/CTLS, and clinical variables were compared between groups.

**Table 1:** Cairo-Bishop definition of laboratory and clinical tumor lysis syndrome.

TLS type	Diagnostic criteria
**Laboratory TLS (≥2/4 criteria)**	Potassium: ≥6 mEq/L or 25% increase from baseline^a^ Phosphorus: ≥4.5 mg/dL or 25% increase from baseline Calcium: ≤ 7 mg/dL, or 25% decrease from baseline Uric acid: ≥8 mg/mL or 25% increase from baseline
**Clinical TLS** **(Laboratory TLS + ≥1/3 criteria)**	Creatinine: > 1.5 times the upper limit of normal or baseline Seizure Cardiac arrhythmia or sudden death

^a^Underlined indicates criteria (relative changes of ≥25%) used in this study.

*Sources*: Cairo MS, et al. *Br J Haematol*. 2010.

### Statistical analysis

Univariable logistic regression analysis was performed to evaluate clinical variables between the normal and LTLS/CTLS groups. Descriptive and comparative statistics were determined for the overall study population and both groups. The normality of continuous variables was assessed using the Shapiro–Wilk test. For non-normally distributed variables, the Wilcoxon rank-sum test was applied for unpaired data and the Wilcoxon signed-rank test for paired data. Categorical variables were compared using Fisher’s exact test. Odds ratios (OR) were calculated for each of the selected risk factors, providing an estimate of the likelihood of LTLS/CTLS development associated with each variable. The OR values were reported with 95% confidence intervals (CIs) to indicate the precision of the estimate. Quantitative data were expressed as mean ± SD from three independent experiments. Statistical significance was set at *P* < 0.05. All analyses were performed using IBM SPSS Statistics.

## Results

### TLS diagnosis and clinical characteristics

Twenty-four dogs with lymphoma satisfied the study’s inclusion criteria, and 10 different breeds (mixed breeds, 6; miniature Dachshund, 4; Shiba, 3; Labrador retriever, 3; Welsh Corgi, 2; toy Poodle, 2; others, 4) were represented in the study population. According to the Cairo-Bishop criteria, among the 24 dogs included in the study, 5 (20.8%) were diagnosed with LTLS, whereas 2 (8.3%) were diagnosed with CTLS. Clinical information for the 17 normal dogs and the 7 dogs with LTLS/CTLS is presented in Tables S1 and S2. Of these 7 dogs, 5 (dogs 1, 2, 4, 6, and 7) developed TLS on day 3, whereas 2 (dogs 3 and 5) developed TLS on day 5. As characteristic clinical information, all of the 7 dogs with LTLS/CTLS initially were treated with L-ASP. Five (dogs 2, 3, 5, 6, and 7) of the 7 dogs showed ≥10% body weight loss between the onset of lymphoma-related clinical signs and initial presentation. Five (dogs 2, 4, 5, 6, and 7) of the 7 dogs had chronic kidney disease (CKD) classified as stage 2 or 3 according to the International Renal Interest Society criteria. Given the absence of severe dehydration, hypercalcemia, and other underlying conditions, acute kidney injury was deemed unlikely. Additionally, 5 (dogs 2, 3, 5, 6, and 7) of the 7 dogs exhibited metabolic acidosis based on VBG analysis. Adverse events observed during the first 8 days after initiation of chemotherapy are presented in Table 2. All chemotherapy-related events were mild (grade 1–2).

**Table 2:** Adverse events within the first 8 days after chemotherapy initiation.

Categories	Term	Grades										Incidence rate (% of all AEs^a^)	
		Normal groups; (*n* = 17)					LTLS/CTLS groups; (*n* = 7)						
		1	2	3	4	5	1	2	3	4	5	Normal	LTLS/CTLS
**Hematologic**	Neutropenia	2										12 %	0 %
**Metabolic**	Increased ALP	1					1					29 %	29 %
	Increased ALT	1											
	Increased AST	1											
	Increased BUN	1											
	Increased Cre	1					1						
**Gastrointestinal**	Anorexia	1	1									41 %	0 %
	Vomiting	1	1										
	Diarrhea	2											
	Hematochezia	1											
**Constitutional**	Lethargy	1										6 %	14 %
	Seizure							1					
**Grand total**		13	2	0	0	0	2	1	0	0	0	75 %	

Abbreviations: AEs = adverse events; CTLS = clinical tumor lysis syndrome; LTLS = laboratory tumor lysis syndrome.

### Patient demographics and risk factors

The demographic characteristics of the normal and LTLS/CTLS groups are described in Table 3. Comparative analysis of clinical variables between the two groups identified significant associations with specific risk factors. The LTLS/CTLS group exhibited significantly higher prevalence of body weight loss compared with the normal group (5 [71%] vs. 3 [18%], *P* = 0.02). The presence of CKD was significantly more common in the LTLS/CTLS group than in the normal group (5 [71%] vs. 3 [18%], *P* = 0.02). The initial administration of L-ASP was strongly associated with LTLS/CTLS development (7 [100%] vs. 7 [41%], *P* = 0.03). Metabolic acidosis was significantly more prevalent in the LTLS/CTLS group than in the normal group (5 [71%] vs.3 [18%], *P* = 0.02). Conversely, no significant differences were identified between the two groups regarding age, body weight, sex, clinical signs, lymphoma stage, or hematologic and biochemical abnormalities.

**Table 3:** Patients demographics between the normal and LTLS/CTLS groups at initial presentation.

Variables		Normal group; (*n* = 17)	LTLS/CTLS group; (*n* = 7)	*P* value (*P < 0.05)
**Age (year): median (range)**		11 (8-16)	12 (9-14)	0.72
**BW (kg): median (range)**		6.7 (3.4-29.5)	7.4 (4.2-24.2)	0.43
**Sex (*n*)**	Castrated male	7 (41 %)	3 (43 %)	1.0
	Spayed female	10 (59 %)	4 (57 %)	
**Clinical signs (*n*)**		10 (59 %)	4 (57 %)	1.0
≥10% BW loss (*n*)		3 (18 %)	5 (71 %)	0.02*
**Chronic kidney disease**	Number of cases	3 (18 %)	5 (71 %)	0.02*
	Bun (mg/dl); median (range)	25.4 (10.3-43.1)	42.8 (9.8-72.4)	
	Cre (mg/dl); median (range)	1.0 (0.5-2.2)	1.8 (0.8-3.0)	
**Stage (*n*)**	III	6 (35 %)	2 (29 %)	0.78
	IV	9 (53 %)	4 (57 %)	
	V	2 (12 %)	1 (14 %)	
**Substage b (*n*)**		10 (59 %)	4 (57 %)	1.0
**First drug (*n*)**	L-ASP	7 (41 %)	7 (100 %)	0.03*
	VCR	10 (59 %)	0 (0 %)	
**CBC**	WBC (/μL); median (range)	15500 (4200-36200)	17600 (5500-28600)	0.49
	Hematocrit (%); median (range)	40 (22-50 %)	38 (26-55 %)	0.62
	Platelet (10^4^/μL); median (range)	40.2 (18-80)	48.8 (22-76)	0.51
**Biochemistry test**	CRP (mg/dL); median (range)	3.6 (0.3-8.6)	2.4 (0.2-10.1)	0.55
	LDH (U/L); median (range)	86 (30-154)	148 (45-267)	0.06
**Metabolic acidosis**	Number of cases	3 (18 %)	5 (71 %)	0.02*
	pH; median (range)	7.39 (7.20-7.42)	7.29 (7.26-7.42)	
	HCO_3_ (mEq/L); median (range)	25.2 (14.8-29.4)	16.8 (13.6-25.3)	
	BE (mEq/L); median (range)	−0.5 (−6.8-0.5)	−4.5 (−6.9-0.2)	

Abbreviations: CBC = Complete Blood Count; CRP = C-reactive protein; CTLS = clinical tumor lysis syndrome; LDH = Lactate dehydrogenase; LTLS = laboratory tumor lysis syndrome; VCR = vincristine sulfate.

### Risk analysis

The ORs further identified four significant risk factors associated with the development of LTLS/CTLS (Table 4). These risk factors included:

1. ≥10% body weight loss (OR, 11.7; 95% CI, 1.5–91.6; *P* = 0.02)
2. Chronic kidney disease (OR, 11.7; 95% CI, 1.5–91.6; *P* = 0.02)
3. Initial administration of L-ASP (OR, 20; 95% CI, 1.0–413; *P* = 0.02)
4. Metabolic acidosis (OR, 11.7; 95% CI, 1.5–91.6; *P* = 0.02)

**Table 4:** Odds ratios for LTLS/CTLS based on four risk factors.

Risk factors	Odds ratios	95 % CI	*P* value (*P < 0.05)
**≥10% body weight loss**	11.7	1.5-91.6	0.02*
**Chronic kidney disease**	11.7	1.5-91.6	0.02*
**L-asparaginase**	20	1.0-413	0.02*
**Metabolic acidosis**	11.7	1.5-91.6	0.02*

Abbreviations: CTLS = clinical tumor lysis syndrome; LTLS = laboratory tumor lysis syndrome.

### Chemotherapy response and outcome

A comparison of the response to chemotherapy between the two groups is presented in Table 5. The ORR after chemotherapy in the entire patient population was 17 of the 24 dogs (71%). Among the seven dogs with LTLS/CTLS, six (86%) achieved CR, one (14%) achieved PR, resulting in an ORR of 100%. In the normal group, four (24%) achieved CR, six (35%) achieved PR, five (29%) achieved SD, and two (12%) achieved PD, resulting in an ORR of 59%. Assessment of treatment responses identified a significantly higher ORR in the LTLS/CTLS group compared with the normal group (*P* = 0.03). However, no significant differences were observed between the groups with respect to PFS or OS.

**Table 5:** A comparison of the clinical outcomes between the normal and LTLS/CTLS groups.

Variables		Normal (*n* = 17)	LTLS/CTLS (*n* = 7)	*P* value (*P < 0.05)
**Chemotherapy response**	CR	4 (24 %)	6 (86 %)	0.03*
	PR	6 (35 %)	1 (14 %)	
	Others (SD + PD)	7 (41 %)	0 (0 %)	
**Progression-free survival (day): median (range)**		207 (24-426)	172 (14-365)	0.46
**Overall survivals (day): median (range)**		312 (36-456)	286 (24-578)	0.83

Abbreviations: CTLS = clinical tumor lysis syndrome; LTLS = laboratory tumor lysis syndrome; PD= progressive disease; SD = stable disease.

## Discussion

Acute tumor lysis syndrome is well-recognized in human cancer patients. Patients with hematologic malignancies are at higher risk of developing ATLS than those with most solid tumors.^1^ Chemotherapy, immunotherapy, corticosteroids, and radiation can precipitate ATLS. Additionally, several clinical factors are associated with increased risk of ATLS in humans.^1^ In a case series involving three dogs with lymphoma, death occurred within 6 to 18 hours after the appearance of clinical signs of ATLS. The onset of these clinical signs ranged from 18 hours to 8 days after treatment.^10^ Laboratory abnormalities included hyperphosphatemia, hypocalcemia, hyperkalemia, azotemia, and severe metabolic acidosis. Necropsy examination of two of the dogs identified generalized microthrombi and infarcts, suggesting that disseminated intravascular coagulation was a terminal event.^10^ In our study, 5 of 24 dogs were diagnosed with LTLS, and 2 were diagnosed with CTLS according to the Cairo-Bishop criteria. Tumor lysis syndrome developed within five days after the initiation of chemotherapy. Of the seven dogs in our study, six exhibited no clinical signs associated with ATLS and required no treatment. One dog (dog 7) experienced seizures, received supportive care from its primary veterinarian, and showed improvement by the next day. These incidence rates reinforce the clinical relevance of TLS as a critical complication in dogs with lymphoma, highlighting the importance of early and vigilant monitoring during the initial phase of chemotherapy.

In hematologic neoplasms, the turnover rate of nucleic acids is increased, resulting in increased concentrations of purines in the blood associated with the lysis of cells. In humans, purines are catabolized in the liver, ultimately producing UA.^11^ In dogs, UA is further oxidized to allantoin in the liver by the enzyme uricase. This difference in purine metabolism prevents most dogs from developing hyperuricemia. In the studies, the blood UA concentrations all were within the normal range in the LTLS/CTLS group. However, Dalmatians and English Bulldogs lack the uricase enzyme, making them more susceptible to ATLS-associated hyperuricemia, and therefore careful monitoring may be necessary.^12,13^ Moreover, comparative analysis indicated that dogs in the LTLS/CTLS group had significantly higher rates of ≥10% body weight loss, CKD, initial administration of L-ASP, and metabolic acidosis, and these factors may align with TLS pathophysiology. Body weight loss is often indicative of advanced disease progression, increased tumor burden, or cancer cachexia. Such metabolic stress may exacerbate TLS risk by promoting a larger release of intracellular contents upon tumor cell lysis. In dogs with CKD, the kidney's ability to excrete K and P is impaired, consequently worsening electrolyte imbalances and increasing the risk of TLS development.^14–16^ In our study, a strong association was observed between the occurrence of LTLS/CTLS and L-ASP administration. Malignant lymphoma cells contain approximately four times the amount of P compared with normal lymphocytes because of increased nucleic acid and ATP demands.^2^ Consequently, the administration of L-ASP, which is highly effective against lymphoma, causes rapid cell lysis, leading to severe hyperphosphatemia followed by hypocalcemia. This feature emphasizes the importance of careful monitoring in dogs receiving L-ASP treatment. Moreover, the presence of metabolic acidosis emphasizes its systemic impact on metabolic abnormalities. These findings suggest that dogs with one or more of these risk factors require increased surveillance during chemotherapy.

Interestingly, the LTLS/CTLS group demonstrated a significantly higher CR rate (86%) compared with the normal group (24%; *P* = 0.02). This finding may be attributed to increased chemosensitivity of tumor cells, resulting in LTLS/CTLS and enhanced chemotherapy response. However, despite the improved CR rate, no significant differences were observed in PFS (median, 172 vs. 157 days; *P* = 0.09) or OS (median, 286 vs. 312 days; *P* = 0.28) between the two groups. These results suggest that although TLS may correlate with a robust initial chemotherapy response, its associated metabolic disturbances and complications may counterbalance any potential survival benefit. This finding emphasizes the importance of effective TLS management to mitigate adverse effects.^17^ Given these findings, clinicians should implement proactive strategies to identify and manage TLS risk factors. Although the small number of published cases of TLS in dogs does not allow definitive conclusions, dogs presenting with substantial body weight loss, CKD, or those scheduled for L-ASP administration should undergo comprehensive pre-treatment evaluation and close post-treatment monitoring. Early intervention strategies, such as careful hydration and electrolyte management, may decrease TLS-related complications and improve overall treatment outcomes.^10,16^ However, our study had a small sample size, which may limit the generalizability of our findings. Although stage was not significantly associated with the occurrence of ATLS, this negative finding may reflect limited statistical power because of cohort characteristics and sample size. Future research with larger cohorts is warranted to further clarify the relative contribution of each risk factor and to explore preventive strategies tailored to high-risk patients.

In conclusion, we identified ≥10% body weight loss, CKD, initial administration of L-ASP, and metabolic acidosis as significant risk factors for TLS development in dogs with lymphoma. These findings emphasize the need for comprehensive risk assessment and preventive strategies to minimize TLS-related morbidity and mortality.

## Supplementary Material

aalaf088_supplemental_table_clean

## Off-label antimicrobial declaration

Authors declare no off-label use of antimicrobials.

## Institutional animal care and use committee or other approval declaration

Authors declare no institutional animal care and use committee or other approval was needed.

## Abbreviations

ATLS: acute tumor lysis syndrome
BE: base Excess
Ca: calcium
CI: confidence intervals
CKD: chronic kidney disease
CR: complete response
Cre: creatinine
CTLS: clinical tumor lysis syndrome
K: potassium
Lac: lactate
L-ASP: L-asparaginase
LTLS: laboratory tumor lysis syndrome
ORR: overall response rate
OS: overall survival
P: phosphorus
PD: progressive disease
PFS: progression-free survival
PR: partial response
SD: stable disease
UA: uric acid
VBG: venous blood gas
VCR: vincristine sulfate
WHO: World Health Organization
