# Supplementary material for: A potential risk factor associated with acute tumor lysis syndrome in dogs with multicentric lymphoma receiving chemotherapy
Source: J Vet Intern Med. 2026 Feb 2;40(1):aalaf088. doi: 10.1093/jvimsj/aalaf088 (PMC12862642; doi:10.1093/jvimsj/aalaf088)
Supplement: aalaf088_supplemental_table_clean [file aalaf088_supplemental_table_clean.docx]

Supplemental Table. 1 Clinical information of normal dogs without LTLS/CTLS

| No | Breed^*1^ | Age  (Year) | BW  (kg) | Sex^*2^ | Clinical signs^*3^ | BW loss^*4^ | Venous  blood gas^*5^ | Stage  (Substage) | Baseline^*6^ |
| --- | --- | --- | --- | --- | --- | --- | --- | --- | --- |
| 1 | **MD** | 12 | 5.3 | CM | Anorexia,Vomiting | Absence | pH: 7.26  PCO_2_: 42.5mmHg  HCO_3_: 14.8mEq/L  Lac:8.4mEq/L  BE: −6.8mEq/L | Ⅳ  (b) | K:5.2 mEq/L  P:5.8 mg/dl  Ca:9.0 mg/dl  Cre:2.2 mg/dl  UC:0.2 mg/dl |
| 2 | Mix | 14 | 12.4 | SF | Lethargy | Presence | pH: 7.35  PCO_2_: 44.4mmHg  HCO_3_: 25.8mEq/L  Lac: 0.7mEq/L  BE: 0.2mEq/L | Ⅴ  (b) | K:3.8 mEq/L  P:5.1 mg/dl  Ca:9.7 mg/dl  Cre:1.3 mg/dl  UC:0.8 mg/dl |
| 3 | MD | 9 | 6.7 | CM | Nothing | Absence | pH: 7.39  PCO_2_: 42.4mmHg  HCO_3_: 24.8mEq/L  Lac: 0.5mEq/L  BE: 0.1mEq/L | Ⅲ  (a) | K:4.3 mEq/L  P:3.6 mg/dl  Ca:10.2 mg/dl  Cre:1.0 mg/dl  UC:0.1 mg/dl |
| 4 | Mix | 10 | 7.4 | SF | Nothing | Absence | pH: 7.40  PCO_2_: 43.5mmHg  HCO_3_: 27.1mEq/L  Lac: 1.2mEq/L  BE: −0.2mEq/L | Ⅲ  (a) | K:4.1 mEq/L  P:2.9 mg/dl  Ca:11.8 mg/dl  Cre:1.2 mg/dl  UC:0.2 mg/dl |
| 5 | LR | 11 | 24.2 | SF | Anorexia  Pyrexia | Absence | pH: 7.41  PCO_2_: 44.5mmHg  HCO_3_: 26.2mEq/L  Lac: 2.1mEq/L  BE: −0.1mEq/L | Ⅳ  (b) | K:5.2 mEq/L  P:4.2 mg/dl  Ca:11.4 mg/dl  Cre:1.0 mg/dl  UC:0.6 mg/dl |
| 6 | Mix | 16 | 4.2 | CM | Anorexia | Absence | pH: 7.2  PCO_2_: 41.9mmHg  HCO_3_: 19.4mEq/L  Lac: 6.5mEq/L  BE: −4.5mEq/L | Ⅲ  (b) | K:5.6 mEq/L  P:5.4 mg/dl  Ca:9.1 mg/dl  Cre:2.9 mg/dl  UC:0.3 mg/dl |
| 7 | **MD** | 11 | 3.8 | CM | Lethargy  Vomiting | Absence | pH: 7.39  PCO_2_: 40.9mmHg  HCO_3_: 25.0mEq/L  Lac: 2.9mEq/L  BE: 0.2mEq/L | Ⅳ  (b) | K:4.0 mEq/L  P:5.3 mg/dl  Ca:9.9 mg/dl  Cre:1.2 mg/dl  UC:0.4 mg/dl |
| 8 | WC | 12 | 8.9 | SF | Nothing | Presence | pH: 7.37  PCO_2_: 41.9mmHg  HCO_3_: 29.4mEq/L  Lac: 2.5mEq/L  BE: −1.2mEq/L | Ⅳ  (a) | K:4.7 mEq/L  P:5.4 mg/dl  Ca:11.3 mg/dl  Cre:2.2 mg/dl  UC:0.7 mg/dl |
| 9 | Mix | 10 | 12.5 | SF | Anorexia Vomiting | Absence | pH: 7.39  PCO_2_: 46.5mmHg  HCO_3_: 26.2mEq/L  Lac: 0.7mEq/L  BE: 0.4mEq/L | Ⅴ  (b) | K:4.3 mEq/L  P:4.6 mg/dl  Ca:10.2 mg/dl  Cre:0.8 mg/dl  UC:0.2 mg/dl |
| 10 | LR | 14 | 29.5 | SF | Lethargy | Presence | pH: 7.42  PCO_2_: 45.5mmHg  HCO_3_: 23.6mEq/L  Lac: 1.5mEq/L  BE: −0.8mEq/L | Ⅳ  (b) | K:4.2 mEq/L  P:3.8 mg/dl  Ca:11.8 mg/dl  Cre:2.0 mg/dl  UC:0.5 mg/dl |
| 11 | TP | 9 | 3.8 | CM | Nothing | Absence | pH: 7.42  PCO_2_: 44.7mmHg  HCO_3_: 27.2mEq/L  Lac: 2.9mEq/L  BE: 0.1mEq/L | Ⅲ  (a) | K:5.0 mEq/L  P:4.1 mg/dl  Ca:10.4 mg/dl  Cre:1.3 mg/dl  UC:0.4 mg/dl |
| 12 | Shiba | 8 | 6.7 | SF | Nothing | Absence | pH: 7.38  PCO_2_: 47.5mmHg  HCO_3_: 24.2mEq/L  Lac: 1.9mEq/L  BE: −0.8mEq/L | Ⅲ  (a) | K:5.3 mEq/L  P:5.6 mg/dl  Ca:9.5 mg/dl  Cre:3.1 mg/dl  UC:0.6 mg/dl |
| 13 | TP | 9 | 3.4 | SF | Lethargy  Vomiting | Absence | pH: 7.42  PCO_2_: 47.5mmHg  HCO_3_: 25.1mEq/L  Lac: 1.4mEq/L  BE: −0.5mEq/L | Ⅳ  (b) | K:4.8 mEq/L  P:5.1 mg/dl  Ca:9.8 mg/dl  Cre:0.5 mg/dl  UC:0.5 mg/dl |
| 14 | Shiba | 12 | 8.3 | CM | Nothing | Absence | pH: 7.36  PCO_2_: 46.9mmHg  HCO_3_: 25.2mEq/L  Lac: 2.8mEq/L  BE: −1.0mEq/L | Ⅳ  (a) | K:4.3 mEq/L  P:4.9 mg/dl  Ca:11.7 mg/dl  Cre:0.8 mg/dl  UC:0.3 mg/dl |
| 15 | **Papillon** | 15 | 3.6 | SF | Lethargy | Absence | pH: 7.41  PCO_2_: 46.5mmHg  HCO_3_: 25.9mEq/L  Lac: 3.2mEq/L  BE: −0.5mEq/L | Ⅲ  (b) | K:4.0 mEq/L  P:2.6 mg/dl  Ca:10.9 mg/dl  Cre:0.6 mg/dl  UC:0.5 mg/dl |
| 16 | Beagle | 13 | 6.4 | SF | Anorexia | Absence | pH: 7.38  PCO_2_: 44.9mmHg  HCO_3_: 27.6mEq/L  Lac: 2.6mEq/L  BE: 0.5mEq/L | Ⅳ  (b) | K:3.9 mEq/L  P:4.2 mg/dl  Ca:10.6 mg/dl  Cre:0.6 mg/dl  UC:0.4 mg/dl |
| 17 | Pag | 10 | 4.3 | CM | Nothing | Absence | pH: 7.21  PCO_2_: 42.9mmHg  HCO_3_: 20.6mEq/L  Lac: 4.8mEq/L  BE: −5.8mEq/L | Ⅳ  (a) | K:4.2 mEq/L  P:4.4 mg/dl  Ca:9.5 mg/dl  Cre:2.5 mg/dl  UC:0.2 mg/dl |

^*1^ MD; Miniature Dachshund, TP; Toy Poodle, LR; Labrador Retriever, WC; Welsh Corgi.

^*2^ CM; Castrated Male, SF; Spayed Female.

^*3^ Clinical signs at initial presentation

^*4^ Greater than 10% body weight loss

^*5^ Reference ranges venous blood gas taken from Tamura J *et al* ^4^. pH (7.382±0.02); pCO_2_ (42.5±2.1 mmHg); HCO_3_ (24.9±1.7 mEq/L); Base Excess (BE −00±1.8 mEq/L).

^*6^ Baseline value before chemotherapy, K; Potassium, P; Phosphorus, Ca; Calcium, Cre; Creatinine; UC; Uric acid.

^*7^ The onset date of LTLS or CTLS and LTLS/CTLS criteria met items.

|  | No | Breed^*1^ | Age  (Year) | BW  (kg) | Sex^*2^ | Clinical signs^*3^ | BW loss^*4^ | Venous  blood gas^*5^ | Stage  (Substage) | Baseline^*6^ | Onset day  of TLS^*7^ |
| --- | --- | --- | --- | --- | --- | --- | --- | --- | --- | --- | --- |
| LTLS | 1 | **MD** | 12 | 5.3 | CM | Nothing | Absence | pH: 7.39  PCO_2_: 40.4mmHg  HCO_3_: 24.2mEq/L  Lac: 2.5mEq/L  BE: 0.2mEq/L | Ⅲ  (a) | K:4.5 mEq/L  P:4.3 mg/dl  Ca:9.5 mg/dl  Cre:1.2 mg/dl  UC:0.2 mg/dl | Day 3;  K:7.1mEq/L  P:6.5mg/dl |
|  | 2 | Mix | 13 | 12.4 | SF | Lethargy  Vomiting | Presence | pH: 7.29  PCO_2_: 43.5mmHg  HCO_3_: 16.8mEq/L  Lac: 8.2mEq/L  BE: −4.5mEq/L | Ⅳ  (b) | K:4.8 mEq/L  P:5.1 mg/dl  Ca:9.8 mg/dl  Cre:2.4 mg/dl  UC:0.1 mg/dl | Day 3;  K:7.4mEq/L  P:6.9mg/dl  Ca:6.9mg/dl |
|  | 3 | Shiba | 9 | 6.7 | CM | Nothing | Presence | pH: 7.30  PCO_2_: 36.9mmHg  HCO_3_: 15.4mEq/L  Lac: 3.8mEq/L  BE: −4.2mEq/L | Ⅳ  (a) | K:4.3 mEq/L  P:3.6 mg/dl  Ca:11.2 mg/dl  Cre:0.8 mg/dl  UC:0.1 mg/dl | Day 5;  K:7.3mEq/L  P:6.2mg/dl |
|  | 4 | Mix | 10 | 7.4 | SF | Nothing | Absence | pH: 7.42  PCO_2_: 39.5mmHg  HCO_3_: 25.3mEq/L  Lac: 7.2mEq/L  BE: −0.5mEq/L | Ⅲ  (a) | K:4.0 mEq/L  P:2.8 mg/dl  Ca:10.8 mg/dl  Cre:2.2 mg/dl  UC:0.2 mg/dl | Day 3;  P:6.6mg/dl  Ca:6.7mg/dl |
|  | 5 | LR | 11 | 24.2 | SF | Lethargy Pyrexia | Presence | pH: 7.28  PCO_2_: 41.9mmHg  HCO_3_: 17.6mEq/L  Lac: 5.5mEq/L  BE: −6.2mEq/L | Ⅴ  (b) | K:5.5 mEq/L  P:4.2 mg/dl  Ca:10.4 mg/dl  Cre:1.6 mg/dl  UC:0.1 mg/dl | Day 5;  K:7.1mEq/L  P:6.5mg/dl |
| CTLS | 6 | MS | 14 | 4.2 | CM | Anorexia  Vomiting | Presence | pH: 7.28  PCO_2_: 42.5mmHg  HCO_3_: 14.2mEq/L  Lac: 5.9mEq/L  BE: −5.8mEq/L | Ⅳ  (b) | K:5.3 mEq/L  P:5.0 mg/dl  Ca:11.3 mg/dl  Cre:1.8 mg/dl  UC:0.3 mg/dl | Day 3;  K:7.5mEq/L  P:6.4mg/dl  Cre:2.8mg/dl |
|  | 7 | WC | 13 | 9.8 | SF | Anorexia  Lethargy | Presence | pH: 7.26  PCO_2_: 45.5mmHg  HCO_3_: 13.6mEq/L  Lac: 6.5mEq/L  BE: −6.9mEq/L | Ⅳ  (b) | K:4.9 mEq/L  P:4.6 mg/dl  Ca:9.5 mg/dl  Cre:3.0 mg/dl  UC:0.2 mg/dl | Day 3;  K:7.2mEq/L  P:6.1mg/dl  Ca:6.3mg/dl  Seizure |

Supplemental Table. 2 Clinical information of dogs with LTLS/CTLS

^*1^ MD; Miniature Dachshund, LR; Labrador Retriever, MS; Miniature Schnauzer, WC; Welsh Corgi.

^*2^ CM; Castrated Male, SF; Spayed Female.

^*3^ Clinical signs at initial presentation

^*4^ Greater than 10% body weight loss

^*5^ Reference ranges venous blood gas taken from Tamura J *et al* ^4^. pH (7.382±0.02); pCO_2_ (42.5±2.1 mmHg); HCO_3_ (24.9±1.7 mEq/L); Base Excess (BE −00±1.8 mEq/L).

^*6^ Baseline value before chemotherapy, K; Potassium, P; Phosphorus, Ca; Calcium, Cre; Creatinine; UC; Uric acid.

^*7^ The onset date of LTLS or CTLS and LTLS/CTLS criteria met items.
